# Report of Surgical Cases at the Buffalo General Hospital, with Clinical Remarks

**Published:** 1874-12

**Authors:** Julius F. Miner


ART. V.—*Report of Surgical Cases at the Buffalo General Hospital, with Clinical Remarks,* by Professor JULIUS F. MINER, M. D. Reported by BERNARD BARTOW, Resident Physician, and W. W. MINER, M. D.

CASE I.—*Syphilitic Hemiplegia.*—W. H. S., aet 38, American. Clerk, by occupation, was brought to the Buffalo General Hospital, Oct. 22d, 1874, in a semi-conscious condition, with symptoms indicating compression of the brain. There was complete paralysis of the left side of face and body; patient was unable to articulate, and could make himself but little understood, except that he had great pain in his head, referable to the frontal and right parietal regions. He was capable of understanding some things when spoken to sharply, but would immediately relapse into his former condition of stupor. The pupils were largely dilated and responded but feebly to the stimulus of light. The bowels were extremely torpid; the bladder did not seem to be involved in the paralysis, though the patient voided his urine involuntarily, but at normal intervals, and in normal quantity. There was a constant inclination on the part of patient to turn, as if to be upon the paralyzed side.

There was no history of injury connected with these symptoms. There was an abundant syphilitic rupial eruption upon the body and limbs.

The imperfect history obtained from his friends was, that he had been attending to his business until within three days of his admission to Hospital; had complained of pain in his head for some days and felt sick, and acted *queer*. The pain increased in severity, and with it, appeared the paralysis and stupor, slight at first, gradually increasing, until they attained their present gravity.

The presence of the tertiary eruption pointed to the syphilitic poison as the indirect cause of the symptoms, evidently giving rise to intra-cranial periostitis, with large effusion.

Soon after his admission he was given, croton oil, gtt. iij, this failing to evacuate the bowels, it was followed by Hydrarg. Chlor. Mitis gr. xv, without however, any effect. Potass Iod. gr, xx. was given every three or four hours. Little benefit followed the use of these agents; the coma becoming profound, the patient dying thirty-six hours after admission.

The case now in the morgue is an instructive one, would be my choice for presentation at clinic. Similar cases present themselves frequently to those largely engaged in treating the disease here represented, while to others they are infrequent, and it is only recently that this form of syphilitic disease has been fully recognized and understood. This patient came in here day before yesterday, partially paralyzed, both pupils dilated, not entirely unconscious, unwilling to have his clothes removed and he spoke little and with impaired utterance.

We were ignorant of his history, and had it not been for the eruption present we should have been unable to determine the cause of the hemiplegia. The eruption determined it to be syphilitic hemiplegia, which may present itself at any period of syphilitic disease. When the paralysis appears early, that is, in the secondary period of syphilis, it is not of so serious a character as when it appears later, as it is then more under the influence of treatment than afterwards. It may occur twenty years after the primary infection. The rupia here present is a tertiary affection, and does not occur within the first year, and we infer this man had syphilis some time ago. Syphilis in its tertiary period causes nodes, effusions of lymph, inflammation of the periosteum of the bones, and when these are inside the skull they naturally produce paralysis of various degrees, it may be affecting the eyelids causing ptosis, the facial muscles, the fifth nerve, or more extended portions of the nervous system. In enumerating these syphilitic nerve affections, I mention—1, nodes; 2, gummata; 3, congestion; 4, paralysis; 5, paralysis *sine materia*. In these last no lesion is to be found, or no change recognized by the microscope in post mortem examination. Paraplegia indicates nodes or other products situated in some portion of the spinal column; this form though serious, does not disable the patient so greatly as hemiplegia often does. Paralysis occurring early in the history of the disease is thought to arise from congestion or inflammation, and it is most signally under the influence of medicine.

I have notes prepared for me by Dr. Brush, of a number of cases which I have had under treatment. The first of these cases is one 29 years of age, contracted syphilis in 1868, had eruption, loss of hair, sore throat, rheumatic pains and other symptoms. In December, 1872, hemiplegia occurred without loss of consciousness, which latter symptoms authors say is valuable in diagnosing the character of the trouble. The patient is now in a greatly improved condition, and pursues without inconvenience his work in a mercantile office. A married person, forty-three years of age, first had syphilis eighteen years since; in June 1873 had ulceration of throat and pains in limbs at night. In November, had recovered from sore throat under use of Iodide of Potassium. In March, 1873, fell from his chair, and found on attempting to rise that his left side was beyond his control, did not loose consciousness. During my illness he was treated by another physician for apoplectic hemiplegia without improvement. In the latter part of May I commenced giving him iodide of potassium with small doses of blue mass, which was followed by rapid improvement. He hardly suspected the cause of his trouble at first, but when asked if he had not had primary syphilis, he replied "yes." He is now well. A colored whitewasher, aged forty-eight, had syphilis at thirty. When first seen in April, 1873, he had paralysis of lower extremities, bladder, etc., gave no history of syphilis, had been treated by two or three physicians for three months previous, but was growing worse. In May and early June, was almost completely paraplegic; he then acknowledged having had the disease. He was put upon iodide of potassium treatment, and is now able to attend to his business, though he walks with a shuffling gait. A young fellow came to me who had loss of vision in his right eye; a gambler with loss of vision in both eyes; a sailor with paralysis of arm; a negro with partial hemisplegia; all due to syphilis. Twenty years ago no well formed idea of the connection of these cases with this cause would have been had. As to the relation of the severity of symptoms to fatal result, I would say that sometimes patients die at once, and post mortem examination fails to reveal any causative lesion. Again cases have been reported, in which pus to the quantity of a pint, has been found in the cranial cavity, the membranes of the brain riddled with perforative ulcers and the lobes of the brain softened; but death had occurred with very slight symptoms of disease. Post mortem examination may show every extensive lesions where the symptoms of disease previous to death were very slight, and *vice versa*. I had no idea of finding my clinical patient dead this morning; the relation of symptoms to severity of disease is very variable. The internal viscera may be affected to cause death secondarily. If you have a patient with any of these troubles you may say that he will probably recover. The functions of tne affected nerves may not entirely be regained, the cicatrices in nerve structure are not converted into normal nerve tissue, but great improvement may generally be expected in these nervous disorders of syphilis, you can arrest these affections by treatment. The early occurring and slight affections of the face, eyes, etc, that recover so speedily under treatment are due to conjestion. Over the secondary and tertiary symptoms you may have very great control. There are only two weapons with which to combat syphilis; you may find more mentioned but they will only serve to pacify or delude both patient and doctor. Mercury and Iodine are the only unfailing remedies in these cases. Tonics, stimulants, hygienic means, etc., are valuable but not specific. Give mercury to affect the system but not to ptyalism. This remedy should be used for a year when commenced in the secondary stage of the affection; patients generally take it three months and then give up medicine till the symptoms recur. Patients will grow fat upon mercury. Between the secondary and tertiary affection there is a period of freedom from trouble, when the tertiary symptoms appear give iodide of potassium in full doses. Potassium iodide with mercury is frequently given; mercury in combination with the former, by inunction or by mercurial vapor bath, but the iodide is the chief reliance. Five gain doses of iodide of potassium may be sufficient, fifteen to twenty grain doses thrice daily are more suitable treatment. Give this to affect the system, then hold up. The matter of the size of dose is at present under observation. An ounce in twenty-four hours can sometimes be taken without harm. One hundred grains a day can be taken in pills without inconvenience. The patient you observe in the ward with extensive syphilitic erosion of the extremities has been taking fifteen grain doses three times daily; is now taking thirty, and with improvement that was not manifested under a treatment of some length with the former dose. Small doses are entirely inoperative, such as many physicians are in the habit of prescribing. I could relate other cases where an improvement which seemed very remarkable, has followed the increase of the dose by those who had given patients a long course of treatment with these small doses. Iodide of potassium has lately been in great demand in the arts; its price has advanced while its quality is subject to adulteration in considerable degree. Recently a decline in price has occurred, but while giving an inferior article, the size of the dose is not a safe guide in producing constitutional results.

